# Supplementary material for: Effects of seven days’ fasting on physical performance and metabolic adaptation during exercise in humans
Source: Nat Commun. 2025 Jan 2;16:122. doi: 10.1038/s41467-024-55418-0 (PMC11695724; doi:10.1038/s41467-024-55418-0)

Effects of Seven Days' Fasting on Physical Performance and Metabolic Adaptation during Exercise in Humans

Kristoffer J. Kolnes[1,2], Emelie T.F. Nilsen[1,#], Steffen Brufladt[1,#], Allison M. Meadows[3,4], Per B. Jeppesen[5], Øyvind Skattebo[1], Egil I. Johansen[1], Jesper B. Birk[6], Kurt Højlund[2], Janne Hingst[6], Bjørn S. Skålhegg[7], Rasmus Kjøbsted[6], Julian L. Griffin[3,8], Anders J. Kolnes[9,10], Stephen O'Rahilly[11], Jørgen F.P. Wojtaszewski[6], Jørgen Jensen[1,*]

[1]Norwegian School of Sport Sciences, Oslo, Norway

[2] Steno Diabetes Center Odense, Odense University Hospital, Denmark

[3]Department of Biochemistry, University of Cambridge, UK

[4]Laboratory of Mitochondrial Biology and Metabolism, National Heart, Lung and Blood Institute, National Institutes of Health, USA

[5]Department of Clinical Medicine, Aarhus University, Denmark

[6]August Krogh Section for Molecular Physiology, Department of Nutrition, Exercise and Sports, University of Copenhagen, Denmark

[7] Department of Nutrition, Division for Molecular Nutrition, University of Oslo, Norway

[8]The Rowett Institute, Foresterhill Health Campus, University of Aberdeen, UK

[9] Section of Specialized Endocrinology, Department of Endocrinology, Oslo University Hospital, Norway

[10] Faculty of Medicine, University of Oslo, Norway

[11] MRC Metabolic Diseases Unit, Institute of Metabolic Science, University of Cambridge, UK

[#]: These authors contributed equally.

*: Corresponding author: Jørgen Jensen, Department of Physical Performance, Norwegian School of Sport Sciences, Post box 4014 Ullevål Stadion, 0806 Oslo, Norway, E-mail: jorgen.jensen@nih.no, Phone number: +47 23 26 22 49, Fax number: +47 22 23 42 20

Supplementary information

Supplementary Figures

Supplementary Figure 1 – Fatty acids at rest and during exercise

Plasma levels of individual fatty acids (FA) before exercise, and after tests of maximal fat oxidation and $\dot{V}O_{2peak}$ before (white bars) and after six days of fasting (grey bars). White circles represent individuals before fasting and black after fasting, with dotted lines to illustrate individual change. Individual FAs were measured by LC-MS and reported as relative values. A-E: Saturated FA with chain length of 14-18. F-M: Unsaturated FA with chain length as reported in figures. Statistical analyses were performed with repeated ANOVA with Tukey's post hoc tests. P-values are marked with asterisks; [*] p<0.05, [**] p<0.01, [***] p<0.001, [****], p<0.0001. N=11

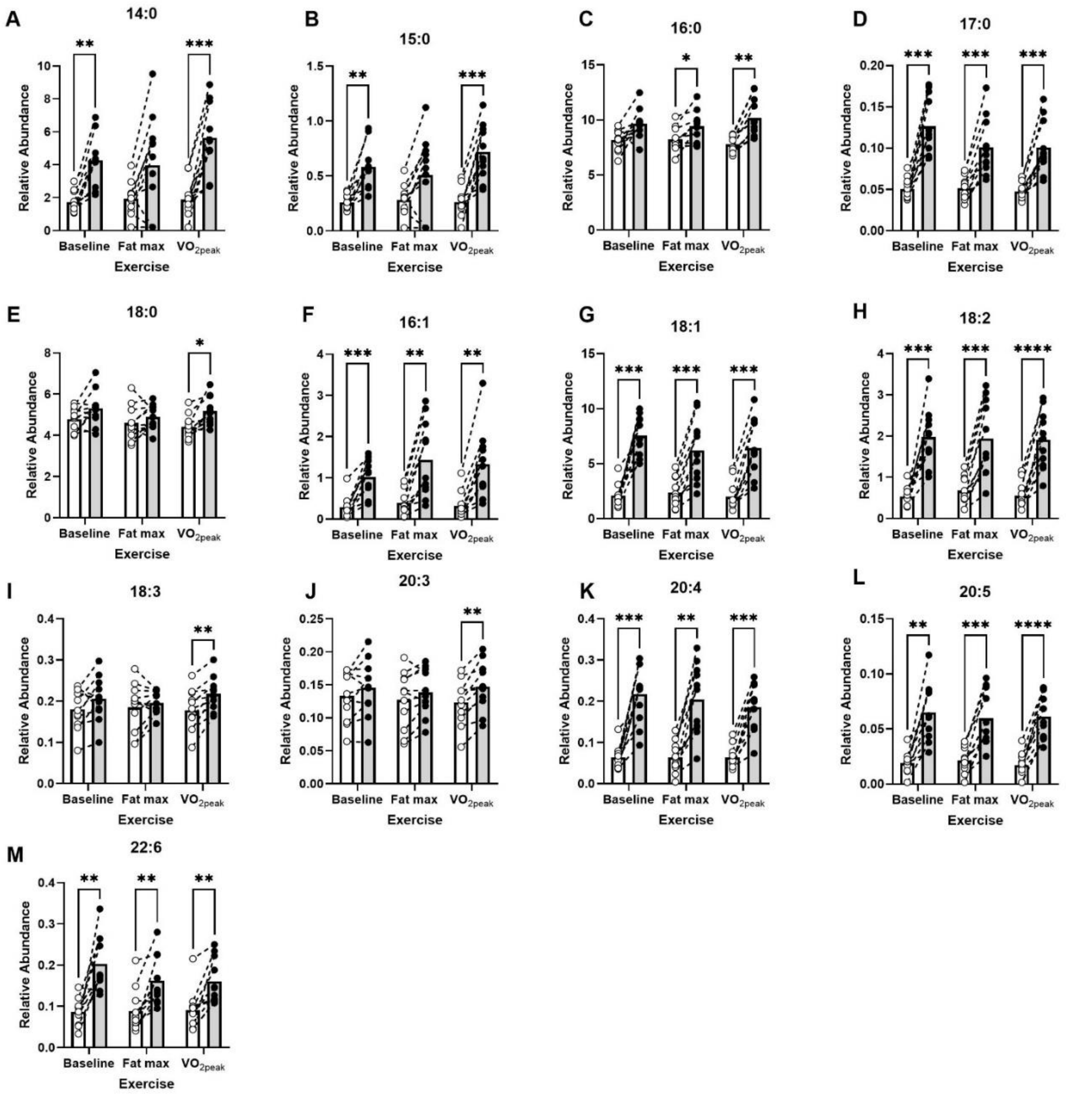

Supplementary Figure 2 – Amino acids at rest and during exercise

Plasma abundance of amino acids before exercise, after maximal fat oxidation test and $\dot{V}O_{2peak}$ test, before (white bars) and after six days of fasting (grey bars). White circles represent individuals before fasting and black after fasting, with dotted lines illustrating individual change. Individual amino acids were measured by LC-MS and reported as relative values: A, B, C, D, E, H, J, and K: Glucogenic amino acids. I, L, M and N: Glucogenic and Ketogenic amino acids. F, G, O and P: Branched chain amino acids and breakdown product. Statistical analyses were performed with repeated ANOVA with Tukey's post hoc tests. P-values are marked with asterisks; [*] p<0.05, [**] p<0.01, [***] p<0.001, [****], p<0.0001. n=11.

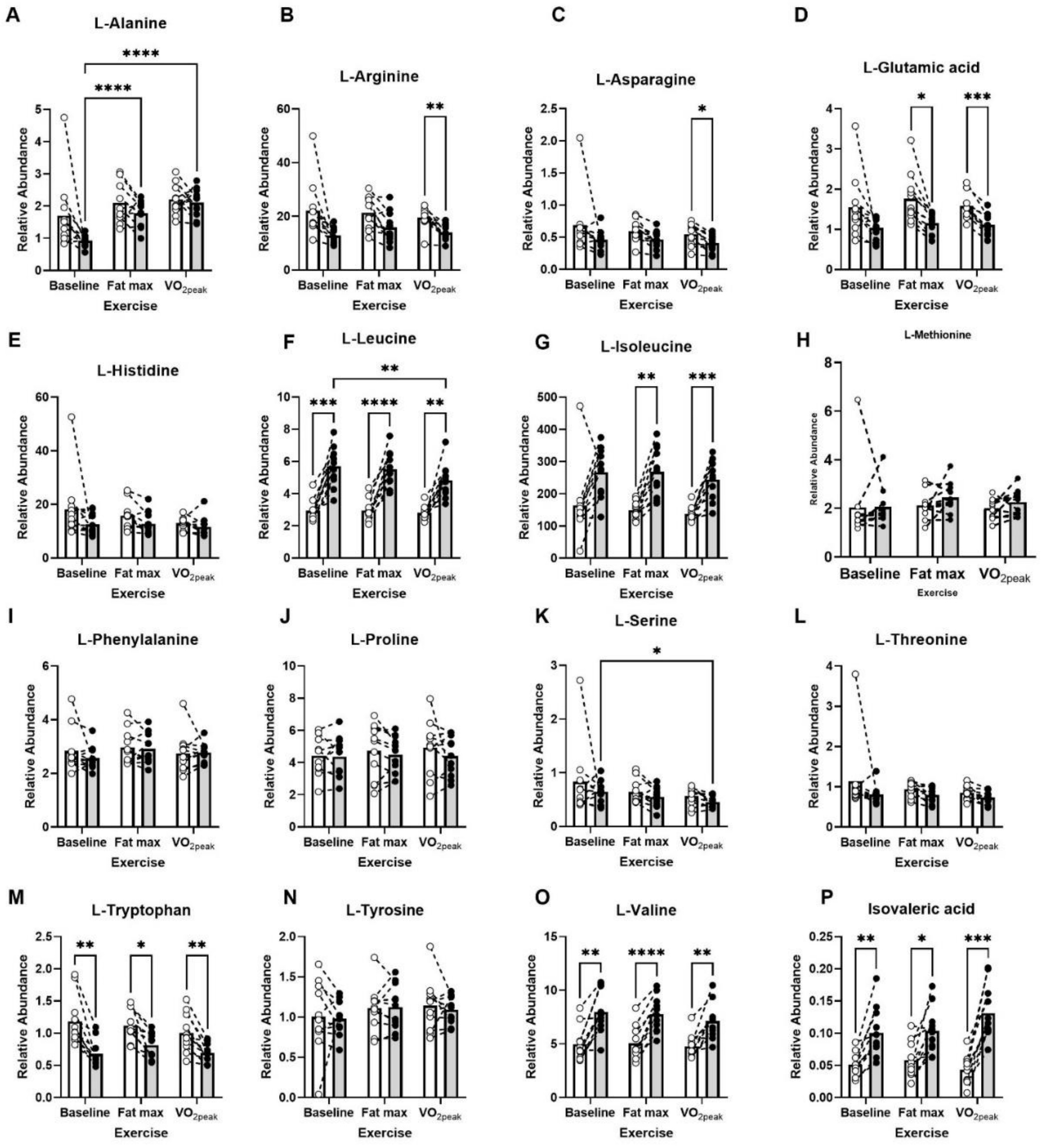

Supplementary Tables

Supplementary Table 1 – Reduction in various body components during fasting

Absolute and relative reduction total body, lean, fat, visceral fat and bone mass for all participants, males and females during the fasting period. Body weight was measured by scale, and mass was estimated from DXA scans.

**Reduction in:**	**All (N=13)**	**Delta-p**	**Males (N=7)**	**Females (N=6)**	**Sex-p**
Body weight (kg)	5.8 ± 0.3	$4.3 \cdot 10^{-10}$	6.4 ± 0.4	5.2 ± 0.3	0.057
Body weight (%)	7.5 ± 0.3	$6.3 \cdot 10^{-11}$	7.0 ± 0.5	8.0 ± 0.5	0.014
Lean mass (kg)	4.57 ± 0.3	$1.1 \cdot 10^{-8}$	5.00 ± 0.3	4.05 ± 0.6	0.165
Lean mass (%)	8.0 ± 0.5	$2.4 \cdot 10^{-9}$	7.3 ± 0.4	8.7 ± 0.9	0.180
Fat mass (kg)	1.43 ± 0.1	$1.7 \cdot 10^{-7}$	1.72 ± 0.1	1.09 ± 0.2	0.011
Fat mass (%)	8.4 ± 1.0	$1.9 \cdot 10^{-6}$	9.2 ± 1.2	7.5 ± 1.7	0.453
Visceral fat (g)	64 ± 27	0.0327	108 ± 38	14 ± 28	0.077
Bone mass (g)	8 ± 10	0.3973	21 ± 9	-6 ± 17	0.18

Data are means ± SEM, for all (13), males (7) and females (6). Delta P-values denote baseline vs after fasting. Two-sided paired t-tests were used for comparison of changes during fasting. Sex-p denote differences between male and females. Two-sided t-tests were used to compare males and females.

Supplementary Table 2: Skeletal muscle expression of selected proteins involved in metabolism. Protein expression levels of metabolic enzymes in skeletal muscle before and after seven days' fasting.

	Before	After	p-value
HK II	100±11	93±11	0.15
Citrate synthase	100±8	97±9	0.48
Mito. Complex I	100±15	123±20	0.29
Mito. Complex II	100±14	93±22	0.79
Mito. Complex III	100±11	111±17	0.51
Mito. Complex IV	100±18	123±22	0.45
Mito. Complex V	100±12	107±17	0.60
FATP4	100±10	119±7	0.19
ACC	100±15	147±21	0.09
AMPK-α2	100±10	107±10	0.52

Protein expression is in arbitrary units. Data are means ± SEM; n=12. P-values for comparison before vs after fasting. Abbreviations, HK II: Hexokinase II; Mito: mitochondrial; Mito Complex 1: Mitochondria electron transport chain I; FATP4: Fatty acid transporter protein 4; ACC: Acetyl-CoA carboxylase; AMPK-α2: AMP-activated protein kinase catalytic subunit alpha 2.

Questionnaire completed by participants every morning during the 7 days' fasting

Questionnaire – VAS (10 cm). The participants marked their answer at the 10 cm scale.

Question 1 (Sp 1): How hungry do you fell? (Not hungry – Extremely hungry)

Question 2 (Sp 2): How strong is the desire to eat? (No desire – Extremely strong)

Question 3 (Sp 3): Which food products do you want the most to eat?

Question 4 (Sp 4): How strong is the desire to eat that product now? (No desire – Extremely strong)

Question 5 (Sp 5): How strong is the desire to interrupt the fast? (No desire – Extremely strong)

Question 6 (Sp 6): How physically tired do you feel? (Not tired – Extremely tired)

Question 7 (Sp 7): How mentally tired you feel? (Not tired – Extremely tired)

Question 8 (Sp 8): How motivated are you to continue the fast? (Not motivated – Extremely motivated)

Question 9 (Sp 9): Do you wish to eat something sweet? (Not at all – Extremely big wish)

Question 10 (Sp 10): Do you want to eat something salty? (Not at all – Extremely big wish)

Question 11 (Sp 11): Do you want to eat something tasty? (Not at all – Extremely big wish)

Question 12 (Sp 12): Do you want to eat something fat? (Not at all – Extremely big wish)

Sleep:

Comments:

Supplementary Figure 3: Representative Western blots. The blots show expression and phosphorylation of glycogen synthase (GS), pyruvate dehydrogenase (PDH) and pyruvate dehydrogenase kinase 4 (PDK4). Blots are from three participants before and after fasting.

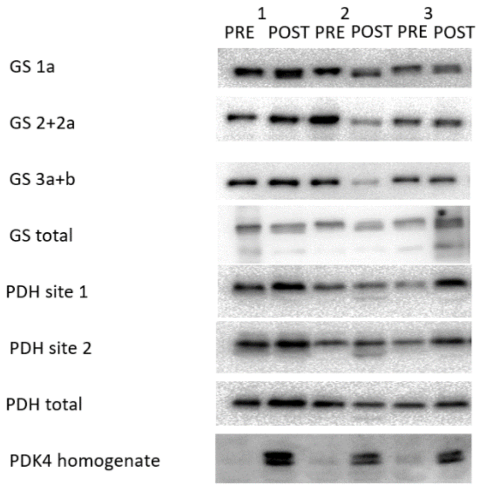

Supplementary Figure 4: **Representative Western blots**. Blots show skeletal muscle expression of some proteins related to metabolism. Blots are from six participants before and after fasting.

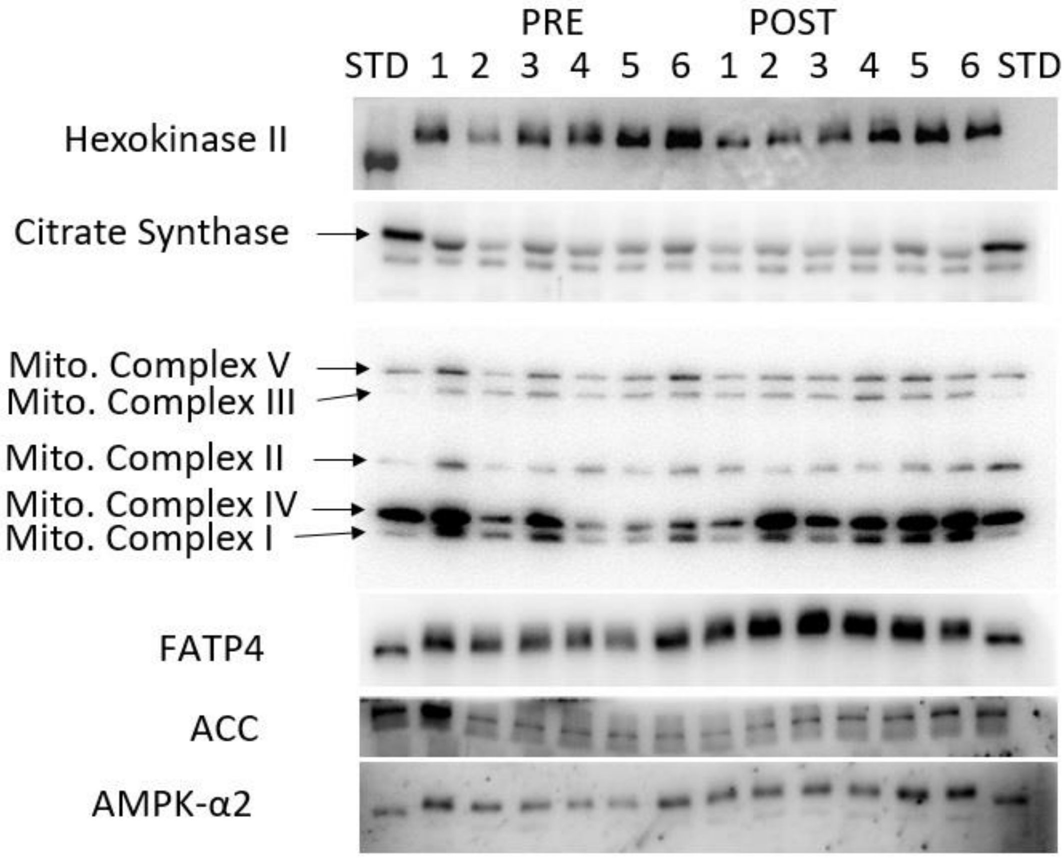

Supplement: Supplementary file 1 — Supplementary Information [file 41467_2024_55418_MOESM1_ESM.pdf]
