# Supplementary material for: Effects of seven days’ fasting on physical performance and metabolic adaptation during exercise in humans
Source: Nat Commun. 2025 Jan 2;16:122. doi: 10.1038/s41467-024-55418-0 (PMC11695724; doi:10.1038/s41467-024-55418-0)
Supplement: Supplementary file 2 — Reporting Summary [file 41467_2024_55418_MOESM2_ESM.pdf]

nature portfolio

Corresponding author(s): DARP NCOMMS-24-50876A

Last updated by author(s): Oct 16, 2024

Reporting Summary

Nature Portfolio wishes to improve the reproducibility of the work that we publish. This form provides structure for consistency and transparency in reporting. For further information on Nature Portfolio policies, see our Editorial Policies and the Editorial Policy Checklist.

Statistics

For all statistical analyses, confirm that the following items are present in the figure legend, table legend, main text, or Methods section.

n/a	Confirmed	
☐	☒	The exact sample size (*n*) for each experimental group/condition, given as a discrete number and unit of measurement
☐	☒	A statement on whether measurements were taken from distinct samples or whether the same sample was measured repeatedly
☐	☒	The statistical test(s) used AND whether they are one- or two-sided<br>*Only common tests should be described solely by name; describe more complex techniques in the Methods section.*
☒	☐	A description of all covariates tested
☒	☐	A description of any assumptions or corrections, such as tests of normality and adjustment for multiple comparisons
☐	☒	A full description of the statistical parameters including central tendency (e.g. means) or other basic estimates (e.g. regression coefficient) AND variation (e.g. standard deviation) or associated estimates of uncertainty (e.g. confidence intervals)
☐	☒	For null hypothesis testing, the test statistic (e.g. *F*, *t*, *r*) with confidence intervals, effect sizes, degrees of freedom and *P* value noted<br>*Give P values as exact values whenever suitable.*
☒	☐	For Bayesian analysis, information on the choice of priors and Markov chain Monte Carlo settings
☒	☐	For hierarchical and complex designs, identification of the appropriate level for tests and full reporting of outcomes
☒	☐	Estimates of effect sizes (e.g. Cohen's *d*, Pearson's *r*), indicating how they were calculated

Our web collection on statistics for biologists contains articles on many of the points above.

Software and code

Policy information about availability of computer code

Data collection	BioRad Image Lab (v. 6.0.1)
Data analysis	Microsoft Excel (Microsoft 365; 2019); PRISM (v. 10.3); Sigma plot (v. 14.0).

For manuscripts utilizing custom algorithms or software that are central to the research but not yet described in published literature, software must be made available to editors and reviewers. We strongly encourage code deposition in a community repository (e.g. GitHub). See the Nature Portfolio guidelines for submitting code & software for further information.

Data

Policy information about availability of data

All manuscripts must include a data availability statement. This statement should provide the following information, where applicable:

- Accession codes, unique identifiers, or web links for publicly available datasets
- A description of any restrictions on data availability
- For clinical datasets or third party data, please ensure that the statement adheres to our policy

We have no permission for making the data public available.

Research involving human participants, their data, or biological material

Policy information about studies with human participants or human data. See also policy information about sex, gender (identity/presentation), and sexual orientation and race, ethnicity and racism.

Reporting on sex and gender	Sex of the participants were self-reported. We did apply the Ethics committee about permission to test sex. Both sex were recruited in rather similar proportion (7 males and 6 females) but we have not disaggregated our data for sex due to low sample size and statistical power.
Reporting on race, ethnicity, or other socially relevant groupings	We do not report on race.
Population characteristics	We do not report population specific characteristics or differences.
Recruitment	Participants were recruited via Norwegian School of Sport Sciences website, via posters and information via social networks. The participants were unpaid and did not receive any compensation.
Ethics oversight	The study was approved by the Ethic Committee at Norwegian School of Sport Sciences (15-220817) and reported to Norwegian Centre for Research Data (NSD: #327898).

Note that full information on the approval of the study protocol must also be provided in the manuscript.

Field-specific reporting

Please select the one below that is the best fit for your research. If you are not sure, read the appropriate sections before making your selection.

☒ Life sciences ☐ Behavioural & social sciences ☐ Ecological, evolutionary & environmental sciences

For a reference copy of the document with all sections, see nature.com/documents/nr-reporting-summary-flat.pdf

Life sciences study design

All studies must disclose on these points even when the disclosure is negative.

Sample size	Sample size was 13. Sample size was calculated from nitrogen secretion and sufficient to find a decline during 7 days fasting.
Data exclusions	No data are excluded from analysis except from metabolomics data. Metabolomics data are from 11 individuals and were analysed using repeated measures two-way ANOVA with Tukey's multiple comparisons test as the post hoc test. Metabolite abundance was normalized to internal standards ("relative abundance"). Metabolites with >20% missing values (below the detection limit) were removed from the dataset. All other missing values were imputed with a 20% minimum value.
Replication	Participants were familiarized with the physical tests. Reproducibility of maximal oxygen uptake and DXA have been validate previously in our laboratory. Replication of the study results were not performed.
Randomization	No randomisation. Pre- and post-test for all.
Blinding	Blinding was not possible since participants completed 7 days fasting.

Reporting for specific materials, systems and methods

We require information from authors about some types of materials, experimental systems and methods used in many studies. Here, indicate whether each material, system or method listed is relevant to your study. If you are not sure if a list item applies to your research, read the appropriate section before selecting a response.

Materials & experimental systems

n/a	Involved in the study	
☐	☒	Antibodies
☒	☐	Eukaryotic cell lines
☒	☐	Palaeontology and archaeology
☒	☐	Animals and other organisms
☒	☐	Clinical data
☒	☐	Dual use research of concern
☒	☐	Plants

Methods

n/a	Involved in the study	
☒	☐	ChIP-seq
☒	☐	Flow cytometry
☒	☐	MRI-based neuroimaging

Antibodies

Antibodies used

Primary antibodies listed with supplier name, catalogue number, clone name / lot number, and dilution: (N/A, not applicable)

HK2, SCBT, sc-130358, J3017, 1 µg/ml
CS, Abcam, ab96600, N/A, 1:1000
OXPHOS Complex 1, Abcam, ab110411, J5383, 1:10000
OXPHOS Complex 2, Abcam, ab110411, J5383, 1:10000
OXPHOS Complex 3, Abcam, ab110411, J5383, 1:10000
OXPHOS Complex 4, Abcam, ab110411, J5383, 1:10000
OXPHOS Complex 5, Abcam, ab110411, J5383, 1:10000
FATP4, Abcam, ab200353, GR3267173-2, 1:1000
ACC, Jackson ImmunoResearch Labs, 016-030-084, 108001, 1:2000
AMPK-α2, SCBT, sc-19131, C0116, 0.2 µg/ ml
GS, Custom-made by prof. Oluf Pedersen, N/A, N/A, 1:40000
p-GS site 2+2a, Custom-made by prof. D.G. Hardie and prof. Jørgen Wojtaszewski, N/A, N/A, 1.5 µg/ml
p-GS site 3a+3b, Custom-made by prof. D.G. Hardie and prof. Jørgen Wojtaszewski, N/A, N/A, 1 µg/ml
PDH E1α, Custom-made by prof. D.G. Hardie and prof. Henriette Pilegaard, N/A, N/A, 1 µg/ml
p-PDH site 1, Custom-made by prof. D.G. Hardie and prof. Henriette Pilegaard, N/A, N/A, 1 µg/ml
p-PDH site 2, Custom made by prof. D.G. Hardie and prof. Henriette Pilegaard, N/A, N/A, 1 µg/ml

Secondary antibodies with supplier name, catalogue number, and dilution
Goat-anti-rabbit-IgG-HRP, Jackson ImmunoResearch Labs, 111-035-045, 1:5000
Goat-anti-mouse-IgG-HRP, Jackson ImmunoResearch Labs, 115-035-062, 1:5000
Rabbit-anti-goat-IgG-HRP, Jackson ImmunoResearch Labs, 305-035-003, 1:5000
Rabbit-anti-sheep-IgG-HRP, Jackson ImmunoResearch Labs, 313-035-003, 1:5000

Validation

Some antibodies have previously been validated in KO cells/tissue. Other antibodies were validated by the manufacturer and confirmed with western blotting by their expected molecular weight or in OE/KO cells and tissue. All Western blot analyses were conducted in the laboratory of JFPW.

AMPK-α2: Validated in PMID: 31010958
HK2: https ://www.scbt.com/p/hxk-ii-antibody-1a7
CS: https://www.abcam.com/en-dk/products/primary-antibodies/citrate-synthetase-antibody-ab96600#
OXPHOS Complex 1: https://www.abcam.com/en-dk/products/panels/total-oxphos-human-wb-antibody-cocktail-ab110411
OXPHOS Complex 2: https://www.abcam.com/en-dk/products/panels/total-oxphos-human-wb-antibody cocktail-ab110411
OXPHOS Complex 3: https://www.abcam.com/en-dk/products/panels/total-oxphos-human-wb-antibody-cocktail-ab110411
OXPHOS Complex 4: https://www.abcam.com/en-dk/products/panels/total-oxphos-human-wb-antibody-cocktail-ab110411
OXPHOS Complex 5: https://www.abcam.com/en-dk/products/panels/total-oxphos-human-wb-antibody-cocktail-ab110411
FATP4: https://www.abcam.com/en-dk/products/primary-antibodies/slc27a4-fatp4 antibody-epr17319-26-ab200353
GS: Validated in PMID: 17928598
p-GS site 2+2a: validated in PMID: 1983
p-GS site 3a+3b: validated in PMID: 32504885
PDH E1α: validated in PMID: 17957032
p-PDH site 1: validated in PMID: 17957032
p-PDH site 2: validated in PMID: 17957032

Plants

Seed stocks

Report on the source of all seed stocks or other plant material used. If applicable, state the seed stock centre and catalogue number. If plant specimens were collected from the field, describe the collection location, date and sampling procedures.

Novel plant genotypes

Describe the methods by which all novel plant genotypes were produced. This includes those generated by transgenic approaches, gene editing, chemical/radiation-based mutagenesis and hybridization. For transgenic lines, describe the transformation method, the number of independent lines analyzed and the generation upon which experiments were performed. For gene-edited lines, describe the editor used, the endogenous sequence targeted for editing, the targeting guide RNA sequence (if applicable) and how the editor was applied.

Authentication

Describe any authentication procedures for each seed stock used or novel genotype generated. Describe any experiments used to assess the effect of a mutation and, where applicable, how potential secondary effects (e.g. second site T-DNA insertions, mosiacism, off-target gene editing) were examined.
